# A Prospective Study on Diabetic Retinopathy Detection Based on Modify Convolutional Neural Network Using Fundus Images at Sindh Institute of Ophthalmology & Visual Sciences

**DOI:** 10.3390/diagnostics13030393

**Published:** 2023-01-20

**Authors:** Awais Bajwa, Neelam Nosheen, Khalid Iqbal Talpur, Sheeraz Akram

**Affiliations:** 1 Ophthalytics, Marietta, GA 30062, USA; 2 Sindh Institute of Ophthalmology & Visual Sciences (SIOVS), Hyderabad 71000, Pakistan

**Keywords:** diabetic retinopathy (DR), fundus images, convolutional neural network, deep learning, ophthalmology

## Abstract

Diabetic Retinopathy (DR) is the most common complication that arises due to diabetes, and it affects the retina. It is the leading cause of blindness globally, and early detection can protect patients from losing sight. However, the early detection of Diabetic Retinopathy is an difficult task that needs clinical experts’ interpretation of fundus images. In this study, a deep learning model was trained and validated on a private dataset and tested in real time at the Sindh Institute of Ophthalmology & Visual Sciences (SIOVS). The intelligent model evaluated the quality of the test images. The implemented model classified the test images into DR-Positive and DR-Negative ones. Furthermore, the results were reviewed by clinical experts to assess the model’s performance. A total number of 398 patients, including 232 male and 166 female patients, were screened for five weeks. The model achieves 93.72% accuracy, 97.30% sensitivity, and 92.90% specificity on the test data as labelled by clinical experts on Diabetic Retinopathy.

## 1. Introduction

Diabetic Retinopathy is the most common microvascular complication of diabetes, and it has become the leading cause of blindness globally. It is estimated that more than 200 million people will be affected by DR in 2040 [1]. The tiny blood vessels (the most light-sensitive area) in the retina at the back of the eye are destroyed by Diabetic Retinopathy. There are two types of DR. One type is Non-Proliferative Diabetic Retinopathy (NPDR), in which the tiny blood vessels swell and leak the fluid that causes a macular edema (swelling of the central part of the retina), which is the dominant cause of mild vision loss. The second type of DR is Proliferative Diabetic Retinopathy (PDR), which is the most advanced class, in which blood vessels grow on the retina’s surface. These cracks in these blood vessels cause vitreous hemorrhages and severe vision loss. The most common risk factors of DR are diabetes (type 1 and type 2), Race (Hispanic and African American people are at greater risk), different medical conditions such as high blood pressure and high cholesterol, pregnancy, and family history. The symptoms include blurred vision, seeing spots and floaters, dark or empty spots in the center of the retina, and decreased vision at night [2].

**Figure 1:**
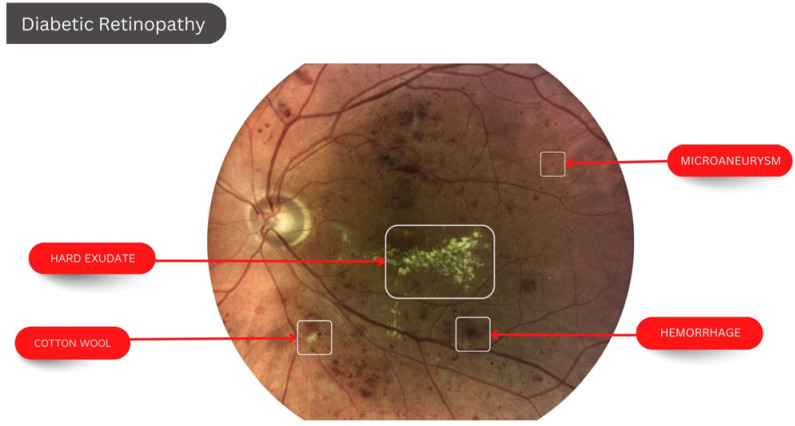
Diabetic Retinopathy.

The detail of Diabetic Retinopathy with various symptoms are shown in Figure 1.

Diabetic Retinopathy (DR) is the most significant cause of blindness among the working-class population in the United States. The prevalence of DR increases with the duration of diabetes, and it causes blindness in the case of late treatment. The CDC estimates that the number of DR patients has increased from 4.06 million to 7.69 million from 2000 to 2010, which is approximate increase of 89 percent. By 2050, this number is expected to double in the United States [3]. According to the National Diabetes survey of Pakistan [4], it has been found that 27.4 million people are affected by diabetes, which is four times higher than it was in 1998. The early detection of DR increases the chances of receiving proper and effective treatment [5]. The symptoms of DR at the initial level are not visible, and the patients do not notice the vision loss until the disease starts to damage their eyes, although this happens in the final phase [6]. The fundus photographs are used for the detection of DR, as these photographs have a high resolution, low cost, and easy storage and transmission.

Ophthalmologists mostly use fundus images to screen for DR manually, which may be biased, complex, and prone to error. It is hard to develop an automatic system consisting of image processing and machine learning approaches to identify DR accurately to overcome these manual errors [7]. Machine learning provides a rapid and practical solution to screening Diabetic Retinopathy. Sambyal et al. [8] presented a method to identify Diabetic Retinopathy using machine learning techniques. The method used various characteristics of two machine learning classifiers, Optimum-Path Forest (OPF) and the Restricted Boltzmann machine. The metrics applied to evaluate the model are accuracy, specificity, and sensitivity. The RBM-1000 achieved the highest diagnostic accuracy in DR screening of 89.47%.

Deep learning is vital in classifying complex images such as the human retina. Khan et al. [9] presented a computational approach for detecting diabetes from ocular scans. The approach classified the retinal images into DR-Positive or DR-Negative ones using deep learning Xception architecture with a dense neural network. The experimental results of the proposed method based on deep learning achieved 96.68% training accuracy and 66.82% validation accuracy.

The remaining part of the paper is organized as follows: Section 2 discusses the related works that have been published in recent years. Section 3 introduces the proposed model based on CNN, and Section 4 contains the experimental results and a detailed discussion of the results. Section 5 concludes the work, and finally, the limitation and future work are discussed in Section 6.

## 2. Related Work

Ali et al. [10] described that the use of deep learning in medical imaging has the potential to improve the accuracy and efficiency of diagnosis and treatment planning, and approaches such as IMNets can be useful tools in this process. However, it is important to note that machine learning systems should be used in conjunction with the expertise and judgement of trained healthcare professionals. Incremental Modular Networks (IMNets) are a specific approach to building deep learning models that involves incrementally adding new modules to the network as needed, rather than building a single, large monolithic network. This can be useful in medical imaging applications because it allows the model to focus on learning specific features or patterns that are relevant to the task at hand, rather than trying to learn everything at once.

A study based on a hybrid deep learning approach [11] classified Diabetic Retinopathy. The proposed method applied a convolutional neural network, VGG16, and VGG19 to extract the features and classify the input images. The model classified the images into five severity level, such as no DR, mild DR, moderate DR, severe DR, and proliferative DR. The APTOS-2019, Messidor-2, and local public DR datasets were used to evaluate the model. The experimental results showed 90.60% accuracy, a 94% F1 score, and 85% recall. Gunasekaran et al. [12] discussed that retinopathy images can be helpful to identify diabetic patients, however, it is a thought-provoking task. To address this challenge, the study used a deep recurrent neural network (RNN) to predict Diabetic Retinopathy from fundus images. The proposed deep learning framework achieved 95.5% precision for the prediction of DR.

Khan et al. [13] implemented various deep neural network architectures such as VGG-net, ResNet, and InceptionV3 with transfer learning. The Gaussian method was applied in the preprocessing phase in order to remove the noise and obtain better results. The dataset consisted of five different classes of DR such as no DR, mild DR, moderate DR, severe DR, and proliferate DR. The results of the multiple deep neural network models showed that InceptionV3 achieved the highest accuracy in training the phase of 81.2% and an accuracy of 79.4% in the testing phase.

Fang et al. [14] presented the DAG network model, consisting of multi-feature fusion of fundus images for the classification of Diabetic Retinopathy. Firstly, three indicative features, retinal hemorrhagic plaque, retinal varices, and fundus neovascularization, were extracted by applying different algorithms. Furthermore, these extracted features were forwarded to the DAG network to learn about the features and fuse many features. Finally, the classification model was applied to classify and identify the DR. The performance of the model was evaluated using a real time dataset collected from a hospital and a DIARETDB1 dataset.

Elloumi et al. [15] presented a novel method to screen DR fundus images captured using a smartphone. The quality of these images can be bad due to the use of handheld devices. To address this issue, the authors applied a NasnetMobile lightweight deep neural network method for feature extraction. A cross-validation process was applied on a structured dataset of 440 fundus images. The model achieved an accuracy of 95.91%, a precision of 95.71%, a sensitivity of 94.44%, and a specificity of 96.92%.

In another study, Kanakaprabha et al. [16] presented a comparative analysis of various deep learning approaches such as CNN, VGG16, VGG19, InceptionV2, ResNet50, MobileNetV2, and Dense Net for the prediction of Diabetic Retinopathy.

The convolutional neural network plays a significant role in the classification of medical images. Sridhar [17] proposed a Diabetic Retinopathy detection system based on a convolutional neural networks architecture. A CNN-based model identified the features of retina fundus images and classified them as DR or no DR and the severity of it. The model was trained on an image dataset that is publicly available on Kaggle. The model achieved impressive results and increased the accuracy in the detection of DR.

Das et al. [18] presented a model for the classification of diabetic Retinopathy based on segmented fundus images features. Morphology and adaptive histogram equalization operations were performed to eliminate the falsely segmented regions. Furthermore, a convolutional neural network was applied to extract the features and for classification using a publicly available DIARETDB1 dataset. The dataset consisted of normal and affected retinas. The experimental results of the proposed model achieved better results compared to those of other traditional schemes. The model produced 97.2% precision and 98.7% accuracy using a standard diabetic retinopathy DIARETDB1 dataset.

Another study by Vives-Boix et al. [19] introduced convolutional neural network-based meta-plasticity to identify Diabetic Retinopathy in fundus images. Synaptic meta-plasticity played a significant role in the backpropagation of every convolutional layer. The model based on an InceptionV3 convolutional neural network was evaluated using a publicly available Diabetic Retinopathy dataset, and it obtained remarkable results in terms of an accuracy of 95.56%, an F1-score of 94.24%, a recall of 90%, and a precision of 98.9%.

Currently, Diabetic Retinopathy classification based on a single-view fundus dataset leads to an inadequate accuracy. Luo et al. [20] addressed this issue and proposed an automatic Diabetic Retinopathy detection system based on a convolutional neural network by integrated multi-view fundus images. The proposed model exploited lesion features from the retinal fundus images, and attention mechanisms were applied to the influential view to achieve a high performance. Furthermore, larger weights were assigned to the significant channels in order to extract the effective features in the network. The proposed model was evaluated using a multi-view Diabetic Retinopathy dataset, and the performance of the model was superior to those of other existing DR detection systems.

Adriman [21] presented a performance evaluation of diabetic retinopathy systems based on various deep learning techniques. The approach consists of two main steps: firstly, local binary patterns (LBP) were applied to extract texture features, and secondly, various deep learning techniques such as ResNet, DetNet, and Dense Net were analyzed for the detection and classification of Diabetic Retinopathy. The experimental results using the APTOS 2019 Blindness Detection dataset indicated that the ResNet, DetNet, VGG16, and Dense Net achieved accuracies of 96.25%, 93.99%, 76.21%, and 84.05%, respectively.

Fatima [22] introduced a unified approach based on a hybrid neural network to detect Diabetic Retinopathy. A novel entropy enhancement approach was applied to enhance the visibility of the medical images, which lead to the extraction of prominent features from the medical images. Afterwards, a hybrid neural network efficiently classified the retinal fundus images. The proposed model was tested using MESSIDOR-2, Ultra-Wide Filed (UWF), Asia Pacific Tele Ophthalmology Society (APTOS) datasets. The extensive experiments were performed to train and validate the proposed model for the classification of Diabetic Retinopathy. Similarly, another study [23] focused on improving the quality of the medical images. The quality of the fundus images was boosted using an Archimedes Optimization Algorithm (AOA) with Kapur’s Entropy (AOA-KE).

Qureshi [24] developed an automatic Diabetic Retinopathy system consisting of the multi-layer architecture of an active deep learning convolutional neural network (ADL-CNN). The system, ADL-CNN, based on a convolutional neural network was used to extract features automatically from the patches and fundus images. Moreover, the regions of interest were segmented, and they described five severity levels in the retinography image. The ADL-CNN model was trained and evaluated using the EyePACS benchmark dataset, and experimental results were compared with state-of-the-art techniques. The performance of the ADL-CNN model is reflected by an accuracy of 98.0%, a specificity of 95.10%, a sensitivity of 92.2%, and an F-measure of 93.0%.

Kalyani et al. [25] introduced deep learning reformed capsule networks to detect and classify Diabetic Retinopathy. In the first step, the convolutional layer and the primary capsule layer were implemented to extract features from the fundus images, and in the second step, the class capsule and SoftMax layer was implemented to predict the specific class of retina images. Extensive experiments were carried out using the MESSIDOR dataset to achieve accuracies of 97.98% for the healthy retinas, 97.65% for the stage 1 ones, 97.65% for the stage 2 ones, and 98.62% for the stage 3 ones.

Gayathri [26] introduced an automatic Diabetic Retinopathy classification system based on a multipath convolutional neural network (M-CNN) and various machine learning (ML) classifiers such as Random Forest (RF), Support Vector Machine (SVM), and J48. The M-CNN was applied to extract the local and global features from fundus images. ML algorithms were used to classify the images, as well as their severity. The performance of the model was evaluated using IDRiD and MESSIDOR datasets with different metrics, such as accuracy, precision, False positive rate (FPR), F1-score, recall, K-score, and specificity.

Bodapati [27] presented an automatic diabetic retinopathy severity classification system based on a composite DNN with a gated-attention mechanism. Multiple pre-trained deep CNNs were applied to obtain the feature descriptors from color fundus retinal images. A spatial pooling approach was applied to obtain a reduced image size without losing much information. The composite DNN model independently acquired these feature descriptors, and this contributed to enhance the model’s performance. The gated-attention block emphasized the lesions of the retinal fundus images. The model was trained and evaluated using the APTOS-2019 Kaggle blindness detection challenge.

Math et al. [28] developed a diabetic retinopathy classification system based on adaptive machine learning. A pre-trained convolutional neural network was applied to obtain various segment levels, and further, all of the segment levels were used to classify Diabetic Retinopathy fundus images. The experimental results of the model obtained 96.37% sensitivity, 96.37% specificity, and 0.963 for the area under the ROC curve using the Kaggle dataset.

Gao [29] introduced a diabetic retinopathy grading system comprised of fundus fluorescein angiography images. Deep learning algorithms such as VGG16, DenseNet, and ResNet50 were applied to the Xian and Ningbo datasets. It was observed that VGG16 achieved best results, with 94.17% accuracy and 0.972 AUC.

Kobat et al. [30] described that DR is a common complication of diabetes that affects the blood vessels in the retina, the light-sensitive tissue at the back of the eye. They applied a pre-trained DenseNET model and divided the digital fundus images into horizontal and vertical patches to detect Diabetic Retinopathy, and the model used two different datasets to validate the performance to detect DR. The model achieved 84.90% accuracy for 10-fold cross-validation.

## 3. Proposed Methodology

**Figure 2:**
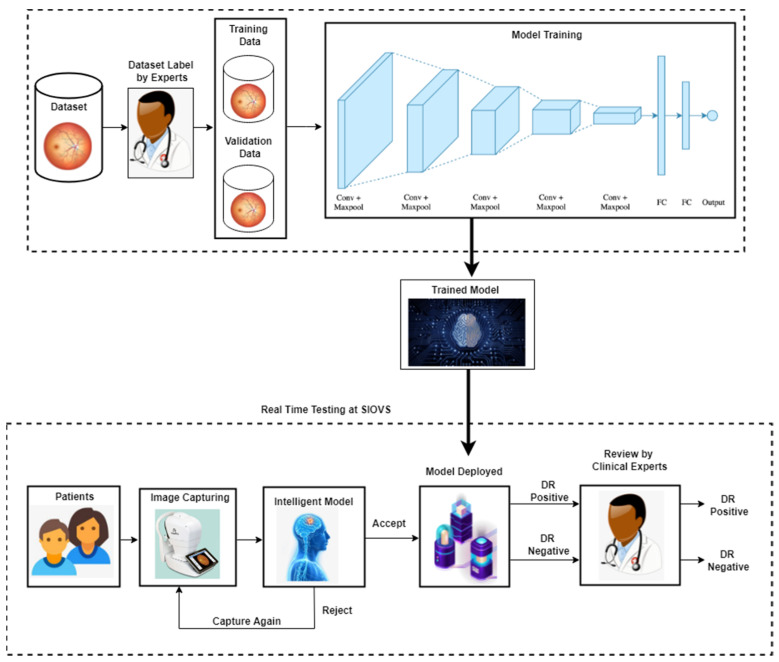
Diabetic Retinopathy detection architecture.

In Figure 2, the overall methodology of the classification of Diabetic Retinopathy architecture is presented. The DR model consists of two phases, the first phase shows the training and validation, and second phase comprises the testing phase. In first phase, the dataset is collected from multiple sources, for example, hospitals and different paid data repositories. The private dataset consists of 57,625 DR images. The dataset is labeled by experts as either DR positive or DR negative. This dataset is further divided into training and validation datasets. The training dataset is used to trained the model, and validation dataset checks the performance of the model during training. The training dataset consist of 80% of the dataset, and validation dataset consisted of 20% of dataset. After splitting the dataset, the dataset is used as an input for the convolutional neural network-based DR model to train and validate the model.

The testing dataset is build using the patients’ data at Sindh Institute of Ophthalmology & Visual Sciences (SIOVS) Hyderabad, Pakistan, by using image-capturing devices.

The DR model consists of convolutional and pooling layers. In the convolutional layers, the convolutional operations were performed with a different filter size to extract the features from the input dataset. The output of the convolutional operations is called the feature or activation map. Furthermore, the Rectified Linear Unit (ReLU) activation function (AF) is applied to convert linear data into non-linear data. After convolutional layer is used, the pooling layer activates. Max pooling, min pooling, average pooling, and sum pooling are common types of pooling layers. In this study, the pooling layer is applied to reduce the size of activation map without losing the important information in the dataset. Subsequently, the convolutional and pooling layers and the feature map are converted into a vector. In the DR model, multiple fully connected layers are used, and finally, the output is obtained from the DR model in the shape of DR-Positive and DR-Negative results.

In the second phase of the study, real-time DR data in the testing dataset are acquired from the patients at the Sindh Institute of Ophthalmology & Visual Sciences (SIOVS) Hyderabad, Pakistan, by using image-capturing devices. A real-time testing dataset consisting of images obtained from patients with Type II diabetes was built for five weeks. The quality of the real-time test dataset images are evaluated by an intelligent model. If the quality of the image is not acceptable according to the intelligent model, then the image is rejected and captured again. If the real-time DR image is accepted by the intelligent model, then the image is evaluated on trained model to classify it into DR-Positive or DR-Negative ones. These classified images are further reviewed by clinical experts who provide their expert opinion in order to evaluate and compare the performances of the trained models.

## 4. Results and Discussion

This study elaborates on the experiments of the proposed model. The model classifies Diabetic Retinopathy into DR positive and DR negative. DR positive indicates that fundus image has Diabetic Retinopathy and the patient needs proper treatment. DR negative means that fundus image has not Diabetic Retinopathy and the patient does not need any treatment.

**Figure 3:**
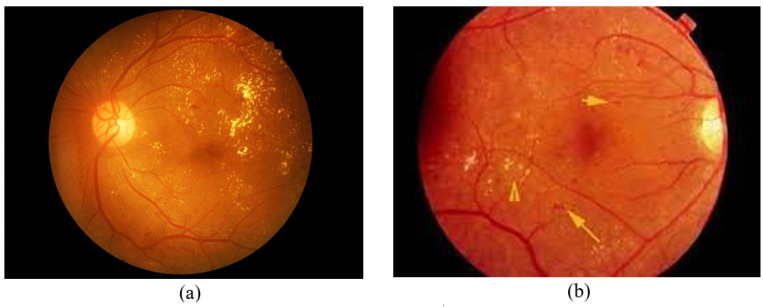
Diabetic Retinopathy images (**a**) DR Negative. (**b**) DR Positive.

In this study, the DR model is trained, validated, and tested on a private dataset. This dataset comprises a total number of 57,625 DR images. The dataset is further divided into 80%, which is used for training, and 20%, which is used for the validation of the DR model. Figure 3a,b shows the DR images.

The statistical performance evaluation metrics such as accuracy [31], sensitivity [32], and specificity [33] as metrics are adopted to compare the overall performance of the DR model. These criteria are given in Equations (1)–(3).

(1) $$Accuracy=\frac{\left(TN+TP\right)}{\left(TN+FN+FP+TP\right)}$$

(2) $$Sensitivity=\frac{TP}{\left(TP+FN\right)}$$

(3) $$Specificity=\frac{TN}{\left(TN+FP\right)}$$

Table 1 represents the confusion matrix of the model for the validation phase. A total number of 11,525 samples are obtained to validate the proposed model. A total number of 11,525 samples are further divided into DR-Negative and DR-Positive ones. The model predicts 5210 (TN) samples as DR-Negative ones correctly as they are actual DR-Negative images, and 614 (FP) samples are incorrectly predicted DR-Positive ones, but they are actually DR-Negative images. The model incorrectly predicts 147 (FN) samples as DR-Negative ones, as these samples are DR-Positive ones. The model correctly predicts 5554 (TP) samples as DR-Positive ones, as they are actually DR-Positive ones. The model achieves 93.40% validation accuracy, 97.42% validation sensitivity, and 89.45% validation specificity.

Table 2 demonstrates the summary of the testing dataset collected during the real-time implementation of the Diabetic Retinopathy model. The data consists of 398 samples, which consists of 232 male and 166 female patients. The average age of the male patients is 49.83 years, and it is 49.71 years for the female patients. The overall average of the patients is 49.76 years.

Table 3 presents the results of Diabetic Retinopathy on the real-time testing dataset consists of five weeks of data obtained from the SIOVS. The total number of samples (398) of Type II are taken to test the Diabetic Retinopathy model. Furthermore, the total number of male patients is 232, in which 196 of them are classified as DR Negative and 36 of them are classified as DR-Positive patients. The total number of female patients is 166, in which 128 of them are classified as DR Negative, and 74 of them are classified as DR-Positive patients.

Table 4 illustrates the results of the images reviewed by the clinical experts, which were classified by the model earlier. The model predicts a total number of 324 samples as being DR Negative, but the clinical experts decided that 301 samples are DR Negative and 23 samples are DR Positive. The clinical experts classified six samples as DR Negative and sixty-eight samples as DR Positive among 74 samples, which are classified as DR Positive by the model. The model achieves 93.72% accuracy, 97.30% sensitivity, and 92.90% specificity after the evaluation of the clinical experts of the Diabetic Retinopathy images.

**Figure 4:**
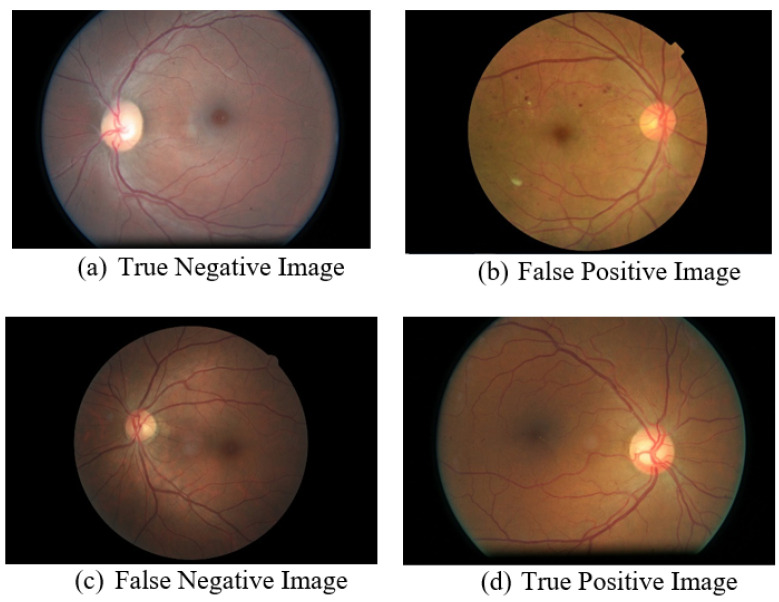
Results of classified images reviewed by clinical experts.

In Figure 4, the sample images of the confusion matrix in Table 4 are shown. The model predicts it to be a DR-Negative image, and the clinical expert also confirmed that image (a) is DR Negative. The model predicts it to be a DR-Positive image (b), but according to the clinical expert, it is DR Negative. The model predicts image (c) as being DR Negative, but as per clinical experts, it is DR Positive. The last image (d) is predicted as being DR Positive by the model, and this is confirmed by the clinical experts.

**Figure 5:**
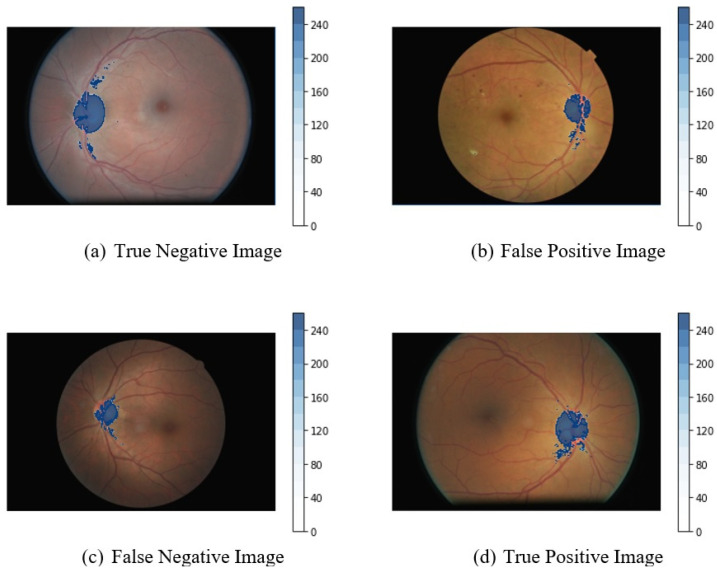
Heat map obtained from last convolution layer are imposed on original image.

In Figure 5, the heat map obtained from the last layer of the convolution layers are imposed onto the original image. The kernel is applied onto a layer to produce feature maps for next convolution layer, and this process continues until it reaches the last convolution layer. The feature maps in the last convolution layers are flattened and used for classification in the deep neural network. The values in the features map of the last convolution layers are converted into range from 0 to 255 in order to impose them onto the original image.

**Figure 6:**
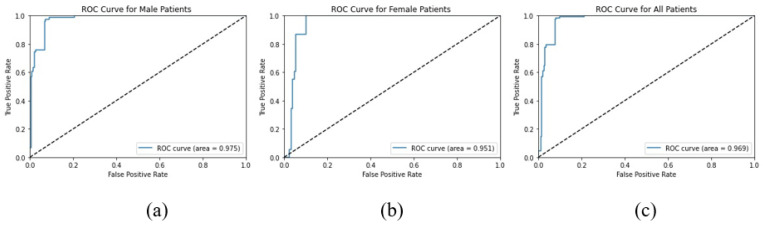
ROC curve of the Diabetic Retinopathy model for (**a**) males, (**b**) females, and (**c**) all of the patients.

In this study, the ROC curve shows the performance of the DR model, and this curve has two parameters: true positive rate and false positive rate. In Figure 6, the ROC curves are plotted for the performance of the Diabetic Retinopathy model on the test data (Figure 6a: Males; Figure 6b: Females; Figure 6c: All Patients) collected at SIOVS. The area under the ROC curve (AUC) for the male patients is 0.975, and for female patients, it is 0.951, and for all of the patients, it is 0.969.

The ROC curve is an important metric to evaluates the performance of the model. The AUC shows that model is clearly classifying samples into positive and negative samples.

## 5. Conclusions

The study initiated by collecting DR datasets from different sources and by clinical experts labeling the dataset. The state-of-the-art deep learning model is developed. The model is trained and validated on the labelled dataset, which achieved an accuracy of 93.40%, a sensitivity of 97.42%, and a specificity of 89.45%. The model was deployed, and the patients were tested in real time at SIOVS. The high quality of the images is ensured by the intelligent system. The images are classified into DR-Positive and DR-Negative ones by the model, which are further analyzed by clinical experts to evaluate the performance of the model in real time. Based on the results of the clinical experts, the model achieves an accuracy of 93.30%, a sensitivity of 97.30%, and a specificity of 92.90%. The value of the AUC for all if the patients and the male and female patients as well shows the impressive performance of the model.

## 6. Limitations and Future Work

The proposed model is based on a modified Convolutional Neural Network (CNN), which needs a large amount of data to train. This model used a fully labelled dataset. In future, the work can extend using semi-labelled and un-labelled datasets. The testing dataset is limited, and it was collected using the same device. In future, the model can be improved and tested on images collected using different types of image-capturing devices.

## Figures and Tables

**Table 1:** Confusion matrix of Diabetic Retinopathy for model validation.

**Actual Label**		**Predicted Label**	
		**DR Negative**	**DR Positive**
	**DR Negative**	True Negative (TN) 5210	False Positive (FP) 614
	**DR Positive**	False Negative (FN) 147	True Positive (TP) 5554

**Table 2:** Summary of data collection at SIOVS.

Gender	No. of Samples	Age (Years) (Mean ± SD)
Male	232	49.83 ± 12.67
Female	166	49.71 ± 11.73
Total	398	49.76 ± 12.1

**Table 3:** Results of Diabetic Retinopathy model for data at SIOVS.

Gender	No. of Samples	DR Negative	DR Positive
Male	232	196	36
Female	166	128	38
Total	398	324	74

**Table 4:** Confusion matrix of Diabetic Retinopathy model after being reviewed by clinical experts.

**Actual Label**		**Predicted Label**	
		**DR Negative**	**DR Positive**
	**DR Negative**	True Negative (TN) 301	False Positive (FP) 23
	**DR Positive**	False Negative (FN) 2	True Positive (TP) 72

## Data Availability

The simulation files/data used to support the findings of this study are available from the corresponding author upon request.
